# Proprotein Convertase Subtilisin/Kexin Type 9: From the Discovery to the Development of New Therapies for Cardiovascular Diseases

**DOI:** 10.6064/2012/927352

**Published:** 2012-09-11

**Authors:** Nicola Ferri

**Affiliations:** Dipartimento di Scienze Farmacologiche e Biomolecolari, Università degli Studi di Milano, Via Balzaretti 9, 20133 Milano, Italy

## Abstract

The identification of the HMG-CoA reductase inhibitors, statins, has represented a dramatic innovation of the pharmacological modulation of hypercholesterolemia and associated cardiovascular diseases. However, not all patients receiving statins achieve guideline-recommended low density lipoprotein (LDL) cholesterol goals, particularly those at high risk. There remains, therefore, an unmet medical need to develop additional well-tolerated and effective agents to lower LDL cholesterol levels. The discovery of proprotein convertase subtilisin/kexin type 9 (PCSK9), a secretory protein that posttranscriptionally regulates levels of low density lipoprotein receptor (LDLR) by inducing its degradation, has opened a new era of pharmacological modulation of cholesterol homeostasis. This paper summarizes the current knowledge of the basic molecular mechanism underlying the regulatory effect of LDLR expression by PCSK9 obtained from *in vitro* cell-cultured studies and the analysis of the crystal structure of PCSK9. It also describes the epidemiological and experimental evidences of the regulatory effect of PCSK9 on LDL cholesterol levels and cardiovascular diseases and summarizes the different pharmacological approaches under development for inhibiting PCSK9 expression, processing, and the interaction with LDLR.

## 1. The Discovery of Proprotein Convertase Subtilisin/Kexin Type 9

Intramolecular proteolytic processing at specific amino acid sites is a common posttranslational modification required for a correct processing and/or activation of precursors proteins into biological active forms. Analysis of human genome has allowed to annotate a total of 553 genes that encode proteases or protease homologues [1]. Proteases are generally classified according to the reaction mechanisms and nature of active site residues involved in the mechanism of proteolysis into serine, cysteine, aspartyl, and zinc (metallo) proteases. The proprotein convertases are serine proteases responsible for the proteolytic processing of a large number of polypeptide hormones, growth factors and their receptor, adhesion molecules, enzymes, and various proteins. This family of proteases is constituted by seven known basic amino acid-specific proteases (PC1/3, PC2, PC4, PACE4, PC5/6, and PC7) and two nonbasic amino acid-specific convertases, SKI-1 and the neural apoptosis-regulated convertase-1 (NARC-1) also known as proprotein convertase subtilisin/kexin type 9 (PCSK9) [2]. PCSK9 was first discovered by Dr. Seidah et al. by searching, with the protein BLAST program, for short conserved segments similarities within the SKI-1 catalytic subunit [3]. This approach was pursued, considering that the presence of processing sites was not recognized by the known proprotein convertases [4]. From the patented database, a putative convertase was identified, previously cloned by two different pharmaceutical companies, named neural apoptosis-regulated convertase 1 (NARC-1; Millenium Pharmaceuticals, Cambridge, MA, Patent no. WO 01/57081 A2) and LP251 (Eli Lilly, Patent no. WO 02/14358 A2).

NARC-1/PCSK9 was then shown to belong to the proteinase K subfamily of subtilases and to be synthesized as a soluble zymogen that undergoes autocatalytic intramolecular processing in the endoplasmic reticulum [5]. PCSK9 encodes a 692-amino acid glycoprotein with an overall domain structure similar to other proprotein convertase family members and includes a signal peptide, a prodomain, a subtilisin-like catalytic domain, and a variable C-terminal domain (termed V-domain) with a fold not previously observed in subtilisin-like serine protease [6]. PCSK9 contains a catalytic triad (Asp^186^, His^226^, and Ser^386^) that superimposes well on the catalytic triads of other subtilisins [3, 7, 8]. PCSK9 processing occurs in the secretory pathway, and the autocleavage generates a stable PCSK9 heterodimer composed of a 14-kDa prodomain fragment and a mature 57-kDa fragment containing the catalytic and C-terminal domains [9]. Accordingly, mutating the conserved serine (Ser386) of the catalytic triad in PCSK9 prevents autocatalytic cleavage resulting in retention of the protein within the endoplasmic reticulum [8, 10]. Coexpression in *trans* of the prodomain and catalytic fragments, either WT or the catalytic dead mutant S^386^A, leads to the secretion of PCSK9 [9]. This evidence further demonstrated the requirement of the autocatalytic processing and a correct association between the prodomain and the catalytic domain of PCSK9 for a proper folding and secretion of the protein. Thus, the prodomain is required for PCSK9 secretion and correct folding thus acting as chaperon molecule for PCSK9 [11–13].

Although, was first reported that the zymogen-processing site of PCSK9 was located at Leu^82^↓ (YVVVLKEETHL, where the underlined L indicates the P1 cleavage position), more specific approaches of microsequencing of the secreted form of PCSK9 from Hek293 and HepG2 cells and of SELDI-TOF analysis permitted to identify the correct cleavage site at SSVFAQ^152^↓ SIP [5]. These results were then confirmed in rat NARC-1 protein [8]. 

Differently from other members of proprotein convertase family, where a second catalytic cleavage is required to release the prodomain and to active the protease [11], no site of secondary cleavage has been identified for PCSK9. Nonetheless, PCSK9 was found to be inactivated by a catalytic cleavage by furin, a member of the proprotein convertase family [14, 15]. Under both *in vitro* and *in vivo* experimental conditions, PCSK9 was found to be cleaved at the RFHR^218^↓ site by furin within an exposed and flexible loop of the catalytic domain [7]. This cleavage leads to unfolding of the protein and detachment of its prosegment [15].

The crystal structure of the native PCSK9 revealed a tightly bound prodomain that is predicted to render the active site inaccessible to exogenous substrates [7, 16, 17]. Indeed, the four C-terminal amino acids of the prodomain (residues 149–152) bind the catalytic site and are further stabilized in this position by an extension at the N-terminus of the prodomain. Nevertheless, some proteolytic activity of PCSK9 has been detected in both total cell lysates and cultured media of primary mouse hepatocytes and immortalized human hepatocytes by using a fluorogenic substrate corresponding to the cleavage site [8, 18]. The same assay was not suitable for plasma samples. The catalytic activity of PCSK9 from cell lysates or conditioned media is approximately 100-fold higher than that of recombinant proteins, suggesting that the prodomain of endogenously produced PCSK9 might leave some access to the substrate groove [18]. The last domain (V-domain) is constituted by three subdomains of *β*-strands each stabilized by three internal disulfide bonds. The particularity of this domain is that is reached in histidine domains. 

Analysis of tissue-specific PCSK9 knockout mice indicated that almost all circulating PCSK9 is derived from the liver [19]. In humans, the plasma levels of PCSK9 vary over a very wide range (~100-fold) among normal, apparently healthy individuals, from a minimum of 33 ng/mL to a 2988 ng/mL [20]. Median levels were found significantly higher in women (517 ng/mL) than in men (450 ng/mL) [20]. A significant proportion of circulating PCSK9 is associated with lipoproteins, including high-density lipoprotein (HDL) and low-density lipoprotein (LDL) but not very low-density lipoprotein (VLDL) [21]. 

Tissue distribution analysis in adulthood and during ontogeny in mouse and rat, by northern blots and in situ hybridization histochemistry, revealed that PCSK9 mRNAs is also present in other tissues including kidney, small intestine and cerebellum [3, 19]. By quantitative real-time PCR (RT-PCR), PCSK9 was also detected in mouse perigonadal fat tissue (at levels ~160-fold lower than in the liver) [22], in mouse aorta (~60-fold less abundant than liver), and in mouse pancreatic islets (~3-fold lower compared to liver) [23]. The presence of PCSK9 in the pancreas was then confirmed by immunohistochemical analysis and to be restricted to pancreating *δ*-cells and absent in *α*- and *β*-cells [23]. In human duodenum and ileum, confocal microscopy analysis revealed that PCSK9 is expressed almost exclusively in the epithelial barrier of both enterocytes and goblet cells [24]. By microarray and real-time PCR analysis, PCSK9 mRNA was also detected, although at low levels, in human atherosclerotic arteries [25], and, more recently, our group reported the detection of PCSK9 protein in human atherosclerotic plaques, by western blot and immunohistochemistry analyses [26]. 

In cultured cells, PCSK9 was shown to be highly expressed in mouse and human hepatocyte (primary hepatocytes and the following cell lines HepG2, McA-RH7777, or HuH7) [27–31], but also from other cell types of different origins. Considering the vascular systems, both human smooth muscle cells [26] and endothelial cells (HUVEC) were shown to express PCSK9 [26, 32], while no detectable amount of PCSK9 was found by RT-PCR analysis in human or mouse monocytes and macrophages [26, 30]. The localization of PCSK9 was also examined in the human colonic, enterocyte-like cell line, CaCo-2, and detected a punctuated staining within these cells mainly present in the apical compartment [24]. 

## 2. PCSK9 Interacts with LDLR and Drives Its Degradation

Autosomal dominant hypercholesterolemia (ADH) is an inherited disorder of lipid metabolism, characterized by a selective increase of LDL particles in plasma (type II a hyperlipoproteinemia), giving rise to tendon and skin xanthomas, arcus corneae, and premature mortality from cardiovascular complications [33, 34]. Goldstein and Brown identified the first gene to be linked to ADH, such as the cell-surface low density lipoprotein receptor (LDLR) [35]. Mutations in other genes can also give rise to a similar phenotype of ADH, for example, familial defective apolipoprotein (apo) B-100 is due to defects in the *APOB* gene, which encodes the ligand for the LDLR [36]. A third gene involved in ADH was then located on chromosome 1p34.1-p32 by studying a French family and 12 additional white families originating from France, Austria, Spain, Belgium, and New Zealand in which the involvement of both *LDLR* and *APOB* was excluded [37]. The same region overlapped that was found to be linked to severe hypercholesterolemia in a Utah kindred [38]. In 2003 Abifadel et al. described two mutations in the PCSK9 gene associated with autosomal dominant hypercholesterolemia [39]. This led to classifying PCSK9 as the third gene associated with familial autosomal dominant hypercholesterolemia, with an incidence estimated to be around 2.3%, together with *LDLR* (incidence ~67%) and *apoB* (incidence ~14%). These two mutations, S127R and F216L, identified in French families, were then shown to be “gain of function” alleles that act in a dominant fashion [5, 40]. The S127R mutation resides between the primary and putative secondary zymogen processing sites of the propeptide, while F216L is located close to the active site, which is at His^226^ [39]. Three “loss of function” nonsense mutations, Y142X, C679X, and R46L, of PCSK9 were then discovered by a genotyping approach conducted in 13,761 subjects (10,045 whites and 3,716 blacks) [41]. Plasma levels of total cholesterol, triglycerides, and LDL cholesterol were significantly lower among subjects harboring a nonsense mutation in PCSK9, but the levels of HDL cholesterol were similar in carriers and noncarriers [41]. 

The direct demonstration showed that the effect of PCSK9 on LDL cholesterol was dependent by the regulation of LDLR expression that was achieved by three independent groups using *in vitro* and *in vivo* experimental approaches. The involvement of PCSK9 in lipid metabolism was suggested by a study conducted with three lines of mice (transgenic for SREBP-1a, transgenic for SREBP-2, and knockout for SCAP) that identified PCSK9 as one of the 33 genes regulated by sterol regulatory element-binding protein (SREBP) transcription factors [42]. Starting from these evidences, Sahng et al. demonstrated that overexpression of PCSK9 both in HepG2 cells or *in vivo,* through adenoviral injection in mice, strongly downregulates LDLR expression without affecting apoB secretion and processing of SREBPs [10]. The fast protein liquid chromatography (FPLC) profile of plasma cholesterol from mice injected with an adenovirus expressing PCSK9 clearly showed an increase of LDL fraction and no effect on HDL; this effect was dependent by the presence of LDLR since no effect on the FPLC profile was observed in LDLR null mice [10]. The dependence of LDLR on the hypercholesterolemic effect of PCSK9 overexpression was then confirmed by other groups [28, 43], although a significant increase of cholesterol LDL fractions in LDLR null mice has also been reported [5]. 

After these experimental evidences the PCSK9 null mice were generated [44]. The PCSK9 null mice showed higher hepatic LDLR expression associated with a significant hypocholesterolemic profile of VLDL, LDL, and HDL subtractions [44]. The determination of radiolabeled LDL clearance showed that the time required to reduce by 50% the ^125^I-LDL was 12 min in PCSK9 null mice and more than 60 min in WT mice [44], suggesting that PCSK9 negatively influences the LDL hepatic uptake in mice. 

 From these evidences it was concluded that PCSK9 affects LDLR expression, but whether this action is mediated by circulating or intracellular PCSK9 was still undetermined. This issue was nicely addressed by using transgenic mice overexpressing human PCSK9 under the apoE promoter parabiosed with WT mice [31]. The results of the parabiosis studies provided a clear evidence that PCSK9 can function in plasma to destroy LDLR in liver. Moreover, Lagace et al. clearly demonstrated, by pulse-chase experiments, that PCSK9 is rapidly (within 2 h) and efficiently processed from the precursor form of 74 kDa to the cleaved mature catalytic fragment of 60 kDa and secreted from the cells [31]. Similar experiments also show that overexpression of PCSK9 does not affect the synthesis of the LDLR in HepG2 cells [30]. PCSK9 was then shown, by coimmunoprecipitation experiments, to interact directly with the LDLR on the cell surface and to be internalized, together with the LDLR, in the late endocytic-lysosome subcellular compartments [31, 45]. The internalization of PCSK9 with the LDLR into an endosomal/lysosomal compartment was required for PCSK9 to reduce LDLR protein levels, since this activity is blocked in the absence of ARH [31, 45–49]. Nevertheless, under more extreme experimental conditions, where PCSK9 was strongly upregulated by adenoviral infections, it was documented that a second intracellular pathway for LDLR degradation by PCSK9 may occur within the post-ER compartments without involving the proteasome or lysosomal cysteine proteases [30]. The intracellular pathway appears to be independent from the presence of functional ARH protein [10, 50]. Thus, up to day two different pathways for PCSK9-dependent LDLR degradation have been described and still need to be determined which is their relative contribution in humans under both pathological and physiological conditions. Natural occurring PCSK9 mutants may also act differently than WT PCSK9. For instance, the gain of function mutants D374Y appears not to be so efficiently secreted by the cells although downregulates the LDLR more efficiently than WT [5, 31].

## 3. Structural Requirements for PCSK9-Mediated Degradation of LDLR 

After it was demonstrated that PCSK9 binds directly to the LDLR on the surface of cells [31], further studies were carried out in order to define which domains of these two proteins were involved in the interaction and how changes of pH alter the complex conformation. By expressing LDLR in Hek-293 cells with deleted portions of each of the ligand binding repeats, and the epidermal growth factor-like repeat-A (EGF), -B, and -C, Zhang et al. discovered that PCSK9 binds to EGF-A domain of LDLR [51]. The N-terminal region of the EGF-A repeat contains a calcium ion-binding site coordinated by the residues of N^295^, E^296^, D^310^, and Y^315^ [52–54]. The residue D^310^ was shown to be required for the binding of PCSK9 to EGF-A.

The binding of PCSK9 with the LDLR was shown to be pH dependent and significantly higher at pH 5.2 compared with neutral pH [51]. The *K*_*D*_ values for the interaction between PCSK9 and LDLR were calculated at different pH [7, 55]. The affinity of PCSK9 proteins, either WT or mutants, for LDLR at pH 5.3 is at least 150-fold higher than that at pH 7.4 resulting in *K*_*D*_ values in the low nanomolar range [7, 55]. VLD and LDL were shown to affect the LDLR binding with PCSK9, although their binding domain is distinct from that of PCSK9, the ligand binding repeats, and the EGF-A domain, respectively [55]. How LDL and VLDL interfere with the affinity of PCSK9 with LDLR still needs to be determined, but it has been hypothesized that LDL may cause a conformational change of the receptor (i.e., allosteric effect) or may reduce the accessibility of PCSK9 to its binding site on the LDLR. 

These results indicate that the levels of circulating LDL may have an effect on the PCSK9-lowering action of LDLR expression. On this regard, it is worth to mention that the binding affinity of PCSK9 for the LDLR is significantly lower (*K*_*D*_ = 170–628 nM) [7, 55] than that of LDL (*K*_*D*_ = 5–25 nM) [56–58], and the human plasma concentrations of PCSK9 (0.3 *μ*g/mL) [59] are much lower than that of LDL (~2.8 *μ*M apoB; ~1500 *μ*g/mL) [60]. Thus LDL appears to have a predominant role for the binding to the LDLR compared to PCSK9, although even in the presence of an excess of LDL, a complete inhibition of PCSK9 binding to LDLR has been observed, suggesting that PCSK9 may be helped by a coreceptor or cell surface molecule for the binding with the LDLR [61]. The LDL and PCSK9 competition for the LDLR binding is even more complicated if we consider that PCSK9 may self-associate to form dimers and trimers which show higher LDLR degrading activity than monomer PCSK9 [21]. 

**Figure 1:**
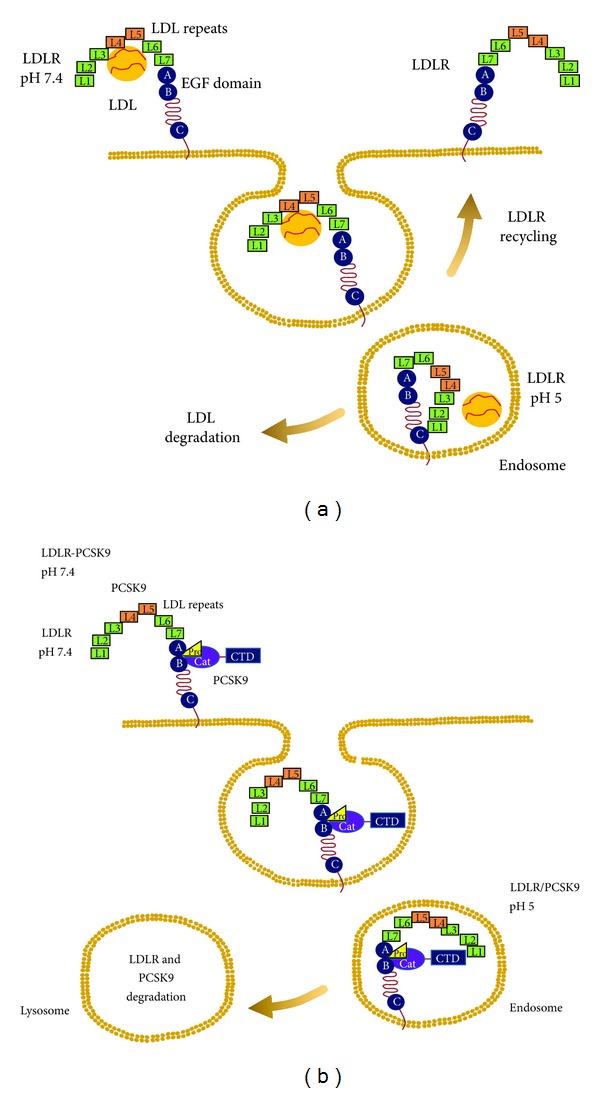
Schematic representation of the PCSK9-mediated LDLR degradation process. (a) On the cell surface at neutral pH, the LDLR can adopt an open extended conformation and binds to LDL predominantly through the L4 and L5 domains of the LDL repeats. At low endosomal pH, LDLR adopts the closed form releasing the LDL and allowing its recycle to the cell surface. (b) Circulating PCSK9 binds the LDLR, restraining its flexibility at the EFG-B-*β*propeller interface. The low pH of the endosomes enhances PCSK9/LDLR affinity, preventing complex dissociation and conformational changes leading to lysosomal LDLR degradation. A possible interaction of the PCSK9 C-terminal domain (CTD) at the endosomal pH has also been described to strengthen the binding between the two proteins.

The crystallization of the extracellular domain of the LDLR at pH of 5.3 provided a possible explanation for how PCSK9 binding might interfere with receptor recycling [62]. The ligand-binding repeats of LDLR are extended at a neutral pH (open conformation), allowing to interact with circulating lipoproteins on the cell surface (Figure 1) [62]. After binding lipoproteins, the receptor-ligand complex is internalized and delivered to an endosome (low pH), where the ligand-binding repeats 4 and 5 form a tight association with the *β*-propeller region of the EGF precursor homology domain (closed conformation), resulting in release of the lipoprotein and delivery of the particle to the endosome. The incubation of the extracellular domain of LDLR with PCSK9 completely inhibited the protein rearrangement observed when the pH shifts from 7.4 to 6.0, while the elution pattern of PCSK9 was not pH dependent [63]. 

The complex PCSK9/LDLR has been crystalized and analyzed in detail [61]. The LDLR showed an extended conformation, with the L7-EGF(A)-EGF(B) region appearing as a stick-like protrusion from the *β*-propeller, with dimensions remarkably similar to those of lipid reconstituted LDLR observed at neutral pH [64] and contrasting with the closed ring-like structure observed at low pH [62]. Consistently with the data reported by Zhang et al. [63], PCSK9 adopted the same structure at any pH values considered [7, 16, 17, 61]. Most of the contacts, at pH 7.0, between PCSK9 and LDLR occurred between the PCSK9 catalytic domain and the LDLR EGF-A domain, resulting in a roughly 1,000 Å^2^  interface [61, 65, 66]. The shift between the extended to the closed receptor conformation is due to a rotation around the residue S^376^, a hinge in the short linker (A^373^VGSIA^378^) connecting EGF-B to the *β*-propeller [61]. Thus, once in the endosomal low-pH milieu, the increased affinity of the PCSK9/LDLR interaction reduces complex dissociation [17, 55, 61], and contacts might form between the LDLR ligand binding repeats and the PCSK9 C-terminal domain (Figure 1) [67]. PCSK9, by holding the LDLR in the extended conformation, interferes with the formation of intramolecular interaction between the L4-L5 subunits and the *β*-propeller thus impeding the release and the recycle of the receptor to the cell membrane (Figure 1). 

Evidences are, thus, consistent with the secreted form of PCSK9 binding directly to the LDLR and resulting in degradation of the receptor within the endosomal/lysosomal compartments, excluding the involvement of the PCSK9 catalytic activity. Indeed, WT PCSK9 and the catalytic dead mutant S386A were shown to reduce, at similar extent, the LDLR expression in HepG2 cells [9]. Furthermore, the addition of the mutation D374Y that improves the binding affinity and the degradation efficiency of PCSK9 on LDLR ameliorates the capacity of the catalytic inactive mutant PCSK9 to degrade LDLR. These evidences further support the conclusion that the binding of PCSK9 to the LDLR facilitates the degradation of the LDLR through a mechanism that does not require proteolytic activity of PCSK9 [9]. 

The overall structure of the EGF precursor homology domain in the VLDLR is similar to that of the LDLR, and, despite these similarities, preliminary studies showed that PCSK9 does not bind to VLDLR in a cell-based assay in COS-M cell type [51]. In contrast, an independent study conducted on three other cell lines (Hek-293, NIH 3T3, and CHO-A7) clearly demonstrated the degradation of both VLDR and ApoER2 by PCSK9 [68]. Different incubation time or cell-type responsiveness, potentially due to a different expression levels of adaptor proteins, such as ARH for the LDLR and Dab1 for ApoER2 and VLDLR [69], may account for this apparent discrepancy. The binding of PCSK9 with these two receptors was then confirmed by using recombinant proteins in the cell-free binding assay (Amplified Luminescent Proximity Homogenous Assay) [70]. The binding of PCSK9 to either VLDLR or LDLR was effectively inhibited by the EGF-A domain, while the interaction with ApoER2 was less affected [70].

## 4. Natural Occurring PCSK9 Variants and Association with Plasma Levels of Cholesterol LDL

The original French family (HC2) harboring a PCSK9 mutation showed a large ADH pedigree comprising three generations [39]. All affected members were characterized by total cholesterol levels above the 97.5th percentile compared to French individuals matched by age and sex. The two probands analyzed were ascertained at age of 17 and 40 years old with 236 mg/dL and 312 mg/dL LDL-cholesterol, respectively [39]. Positional cloning analysis of the same French families led to the identification of the first mutation of PCSK9 (S^127^R) associated with ADH [39]. Through a systematic bidirectional sequencing of the 12 exons of *PCSK9* in 22 probands with ADH, a second mutation was identified (amino acid substitution F^216^L) [39] (Table 1). Both mutations were thus categorized as gain of function mutants. 

The mechanism by which the PCSK9 (S^127^R) protein results in hypercholesterolemia has been suggested to be mediated by an intracellular binding with LDLR [78], because this amino acid substitution delays autocatalytic cleavage and secretion [5], and there is only a modest increase in affinity for the LDLR [7, 55]. However, more recent evidences indicate that PCSK9 (S^127^R) primarily functions through a higher binding affinity to the LDLR on the cell surface, increasing its degradation [88]. Differently, the F^216^L mutation bounds LDLR with similar affinities to those of wild-type PCSK9 at both pH values [7], indicating that there exist multiple mechanisms for various gain of function mutants to enhance the activity of PCSK9. In regard to this, the F^216^L mutant has been shown to result in partial loss of furin/PC5/6A processing at the motif RFHR^218^↓ that leads to inactivation of PCSK9 [14, 15]. However, it cannot be excluded that, aside from the relative resistance to furin processing, the hypercholesterolemia phenotype associated with the F^216^L mutation could also be caused by other as yet undefined properties of this particular mutant. 

Two additional mutations of PCSK9 (D^374^Y and N^157 ^K) were then identified in Norwegian subjects with clinical diagnose of familial hypercholesterolemia [84], and the same mutation was observed in a Utah pedigree [83]. PCSK9 D^374^Y has been extensively characterized and represents the best known gain of function mutant of PCSK9. Its gain of function property results from the higher binding affinity to the LDLR at either physiologic (pH 7.4) or low pH (pH 5.0) [7, 31]. Differently, the reason of the gain of function effect of the PCSK9 N^157 ^K mutant still needs to be determined [5].

To strength the role of PCSK9 on cholesterol homeostasis, additional genomic analysis was performed in order to identify inactivating (loss of function) mutations in patients with low plasma levels of LDL cholesterol [80]. The subjects were from the Dallas Heart Study, a multiethnic probability-based population sample of Dallas County (52% African American, “black,” 29% European American, “white,” 17% Hispanic and 2% other ethnicities) [89]. The DNA sequencing identified two nonsense mutations, the substitution C^426^G and C^2037^A in the African Americans subjects. Both mutations introduce stop codons at residue 142 (Y^142^X) and at residue 679 (C^679^X) [80] (Table 1). The Y^142^X mutations appear to be present only in the African Americans with an incidence of 0.4%, while no other ethnic group considered in the study harbors the same mutation. The C^679^X mutation was found prevalently in the African Americans (1.4%) and less frequently in European Americans (≤0.1%) and in Hispanics (≤0.2%). Similar incidence of these mutations was also observed in a different population sample of African Americans [80]. Interestingly, several healthy persons who are homozygous for PCSK9 null alleles have been described, and one of these persons carrying the two loos of function mutations, Y^142^X and ΔR^97^, having 14 mg/dL of LDL cholesterol level, is healthy and has given birth to two healthy children [75]. The clinical characteristic of subjects with these nonsense mutations showed no effect on triglyceride and HDL cholesterol levels but a significant reduction of LDL cholesterol, although not all subjects harboring these mutations were hypocholesterolemic. In the same study, Miettinen et al. measured the plasma levels of lathosterol (marker of cholesterol biosynthesis) and campesterol (marker of cholesterol absorption) [90]. The presence of PCSK9 mutation was not associated with significant changes on both markers, indicating that PCSK9 mutants do not lower circulating cholesterol levels by reducing cholesterol absorption or synthesis. The same authors extend the study by using DNA sequencing and chip-based oligonucleotide hybridization in order to identify additional variations of PCSK9 that contribute to differences in LDL cholesterol levels [82]. Among black subjects, two missense mutations (L^253^F and A^443^T) were associated with low LDL cholesterol levels, while the H^553^R mutation was accompanied by increased plasma levels of LDL cholesterol. All these mutations were very rare or even absent in the white population. All the mutations associated with low LDL cholesterol did not induce hepatic triglyceride content, as determined using proton magnetic resonance spectroscopy [91, 92].

An additional loss of function mutation of PCSK9 gene was then discovered from Italian patients affected by hypobetalipoproteinemia [74]. One patient had a single nucleotide deletion in exon 1 (c.202delG), which caused a frameshift in mRNA, leading to a premature stop codon (Ala^68^fsLeu^82^X). Two additional subjects, harboring the same mutation, were also identified from the same geographic origin whose mean levels of plasma total cholesterol and LDL-C were similar to those found in familial hypobetalipoproteinemia patients. They were all characterized by very low levels of plasma LDL-cholesterol, supporting the loss of function property of this mutation [74]. Restricted to Japanese population, the mutation R^93^C was associated with low LDL cholesterol levels, suggesting the acquisition of a loss of function property [73]. This mutation is present in the prodomain of the molecule, and how it affected the functionality of PCSK9 still needs to be determined. Instead, a better characterized loss of function mutation (S^462^P) has been identified in Norwegian subjects [84, 93]. The acquired loss of function characteristic of this mutant was due to its retention into the endoplasmic reticulum, even though it does undergo autocatalytic cleavage [93]. Surprisingly, the mutation was identified in a subject with hypercholesterolemia who inherited the mutation from her father, clearly hypocholesterolemic, consistent with the mutation being a loss of function. Instead, her mother had hypercholesterolemia, suggesting that the subject inherited an unknown genetic defect from the mother which caused hyper-cholesterolemia. The counteracting effect of multiple genetic mutations and/or polymorphism on the LDL cholesterol level has been recently observed in a study set out to explore the prevalence and effect of mutations in *APOB*, *PCSK9*, *ANGPTL3*, and *LDLR* in carriers of pathogenic autosomal dominant hypercholesterolemia with unexpected low LDL cholesterol levels [94]. Interestingly, the loss of function mutation R46L was shown to be sufficient to significantly compensate the hypercholesterolemia induced by *LDLR* mutations 313 + 1G > C/313 + 2T > C (2.95 versus 6.11 ± 1.76 mmol/L, *P* = 0.013) [94]. 

The similar situation was also observed with a subject harboring the loss of function mutations N^354^I with abolished autocatalytic cleavage of PCSK9 and determining its retention in the endoplasmic reticulum [79]. These evidences clearly demonstrated the importance to determine, by using *in vitro* and *in vivo* experimental models, the functional consequences of novel mutations, without simply combining the mutation with the clinical lipid parameters. Together with the S^462^P, also two additional mutations have been shown to be retained in the endoplasmic reticulum and to act as loss of function mutants, such as the G236S [79] and the C679X [75]. It is likely that all three mutations do not undergo a proper folding and thus do not enter into the secretory pathway.

After these evidences, additional allelic variants have been identified and partially characterized in terms of functionality (Table 1). The complete list of independent PCSK9 variants associated with a particular disease has been collected and made available by Dr. Leigh et al. at the University College London (http://www.ucl.ac.uk/fh/). A total of 101 unique allelic variants of PCSK9 have been reported to date [95]. Among these, the majority (*n* = 73) are exonic, 21 intronic, and 7 in the 5′ or 3′ untranslated regions. In the reported data are indicated 27 gain of function variants, 5 of which need to be confirmed and 21 loss of function mutations including the mutations that block the translation of the protein. The remaining variants are either common polymorphisms or associated with a phenotype that still needs to be investigated. 

## 5. PCSK9 and Cardiovascular Diseases

Cardiovascular diseases are the most common causes of mortality, worldwide, accounting for approximately 30% of all deaths [96]. Of the nine common risk factors for cardiovascular diseases, dyslipidemia (defined as an elevated ratio of apolipoprotein B: apolipoprotein A1) is one of the most significant, accounting for 50% of the population-attributable risk for myocardial infarction [96] and 25% of the population-attributable risk for stroke [97]. The effective management of dyslipidemia is therefore essential for the reduction of cardiovascular risk. Lipid metabolism can be disturbed in different ways, leading to changes in plasma lipoprotein function and/or levels. This by itself and through interaction with other cardiovascular risk factors may induce the development of atherosclerosis and increase the incidence of cardiovascular diseases [98]. LDL cholesterol remains the primary target of therapy in most strategies of dyslipidemia management. The most recent Cholesterol Treatment Trialists' Collaboration (CTT) meta-analysis of several trials involving >170,000 patients confirmed the dose-dependent reduction in cardiovascular diseases with LDL cholesterol lowering [99]. This analysis estimated that for each mmol/L (~40 mg/dL) reduction in LDL cholesterol, obtained with statin therapy, a corresponding 22% reduction in cardiovascular mortality and morbidity is achieved [99]. 

Although the inverse relationship between the LDL cholesterol levels and incidence of coronary heart disease (CHD) has been well defined, the role of PCSK9 within this context began to be delineated by studying the population harboring loss or gain of function mutants. The first study aimed at defining the role of PCSK9 on the incidence of CHD was carried out in black subjects having the loss of function mutations Y^142^X and C^679^X [41, 80] and white subjects with PCSK9 R^46^L mutants. The first two nonsense mutations are associated with a 40% reduction in mean LDL cholesterol, while the last with a 21% decrease [80, 82]. The study of these identified populations provided the opportunity to analyze the effects of specific, lifelong reduction in LDL cholesterol levels on the risk of CHDs. This ARIC study was initiated in 1987, and the reported results included the cardiovascular events up to January, 2003 [41]. During the 15-year-follow-up period, the 9.7 percent of black subjects who did not have a nonsense mutation experienced a coronary event. In contrast, only 1 out of 85 subjects (1.2%) with a non-sense mutation of PCSK9 developed CHD [41]. The calculated hazard ratio for CHD among carriers as compared with noncarriers, after adjustment for age and sex, was 0.11 (95 percent confidence interval, 0.02 to 0.81; *P* = 0.008). The incidence was still significantly low also after taking into consideration the blood pressure and the diabetes. Similarly to what was observed with the non-sense mutations, the white subjects homozygous for the R^46^L mutation had a significant reduction of plasma levels of total cholesterol (9%) and LDL cholesterol (15%). A slightly higher mean plasma level of LDL cholesterol was observed in heterozygous patients; thus, the two populations were pooled. Despite the moderate reduction of LDL cholesterol in patients with R^46^L mutation, a still significant protection against CHD was observed (6.3% versus 11.8%). The hazard ratio for CHD among PCSK9 R^46^L carriers relative to non-carriers, after adjustment for age and sex, was 0.5 (95 percent confidence interval, 0.32 to 0.79; *P* = 0.003). Interestingly, the carriers and the non-carriers for the three PCSK9 allelic variants showed a significant reduction of intima-media thickness, a surrogate measure of coronary atherosclerosis (for the Y^142^X and C^679^X: 0.70 ± 0.13 mm versus 0.73 ± 0.16 mm; for the R^46^L: 0.71 ± 0.16 versus 0.73 ± 0.18 mm). From this analysis it was concluded that an LDL cholesterol reduction, due to a loss of function mutations of PCSK9, has a protective effect on CHD even higher than that predicted from LDL lowering trials [100–102]. The strong protection for cardiovascular events thus considered the results of a lifelong reduction in plasma LDL cholesterol in carriers compared to non-carriers, despite the very high prevalence of other nonlipid-related cardiovascular risk factors [41]. Indeed, the more than one-half of the black participants in the ARIC study had hypertension, almost one-third smoked, and nearly 20 percent had diabetes. Nevertheless, the extent of the cardiovascular protection observed in subjects with PCSK9 loss of function mutants has raised the possibility that R46L-encoding allele of PCSK9 reduces CHD by a mechanism unrelated to the LDL lowering effect. A second study that may open to this possibility was carried out in patients with autosomal dominant hypercholesterolemia attributable to PCSK9 gain of function mutation. A 30-year-followup was performed in 4 unrelated white British families comprising 13 affected individuals with the D^374^Y mutation of PCSK9 and compared to familial hypercholesterolemia due to 3 specific mutations in LDLR. PCSK9 patients had significant higher serum total cholesterol concentrations (13.6 ± 2.9 versus 9.6 ± 1.6 mmol/L; *P* = 0.004) [103] and appeared to be more resistant to statin therapy compared to heterozygous familial hypercholesterolemia patients with known mutations in LDLR. Possibly as a consequence of these two phenotypic features, the PCSK9 patients were affected at a much earlier age by premature CHD (35.3 ± 4.8 versus 46.8 ± 8.9 years; *P* = 0.002) [103]. The reason underlying this severe phenotype associated with the PCSK9 D^374^Y mutation is not yet understood and has been suggested to be related to a possible positive role of PCSK9 on apoB secretion [103]. The effect of PCSK9 on apoB secretion was recently reported in an experimental model based on adenovirus overexpression of WT PCSK9 in the liver [27] and was demonstrated to be independent by the LDLR. A direct binding of PCSK9 to apoB was then suggested to protect apoB from the intracellular degradation via the autophagosome/lysosome pathway [27]. 

Thus, putative gain or loss of function mutants were found to correlate with increased or reduced plasma LDL levels and cardiovascular risk, respectively. A genome-wide association study further bolstered the importance of PCSK9 by establishing a linkage between a single nucleotide polymorphism at a locus near PCSK9 with early-onset myocardial infarction [104]. Similar results have also been confirmed in South Asian and Lebanese populations [105–107]. 

## 6. PCSK9 and Experimental Atherosclerosis

Although the regulatory pathways and enzymes involved in cholesterol homeostasis in mice are dramatically different than humans, some relevant information using this experimental model have emerged during the last decade. The first evidence of the effect of PCSK9 to induce LDLR degradation was derived from the hepatic PCSK9 overexpressing by injection of adenovirus encoding for PCSK9 [5, 10, 28]. PCSK9 determined a specific increase of LDL cholesterol without a significant effect on the HDL fraction [28]. Surprisingly, the injection of adenovirus encoding for two gain of function mutants of human PCSK9 (S^127^R and F^216^L) reduced the LDLR protein levels to similar extent than WT PCSK9 [10]. This apparent discrepancy of the data compared to what has been observed in humans carrying these mutations is probably the results of a dramatic overexpression of PCSK9 after adenoviral injection. Both of these studies conducted the plasma lipid analysis 3 days after injection of adenovirus, while at a longer time point (7 days), Benjannet et al. reported a significant increase of LDL cholesterol also in LDLR null mice [5]. The LDLR-independent effect of PCSK9 was suggested to be mediated by a positive effect on apoB production and secretion of PCSK9 [5], although some contrasting results of this parameter have been reported [10]. Daily administration for 3 days of various doses of human recombinant PCSK9 from 3 to 300 *μ*g/mouse showed a dose-dependent reduction of hepatic LDLR associated with increased plasma levels of LDL cholesterol fraction with a minor effect on HDL [45]. Taken together all these experimental data from *in vivo* models clearly documented a direct effect of circulating PCSK9 on hepatic LDLR expression and thus plasma cholesterol levels. 

In 2005 the first paper with the PCSK9 null mice was published [44]. Mating between PCSK9^+/−^ mice produces offspring in the expected Mendelian ratio. PCSK9 null mice are normal in appearance with similar body and liver weights compared to littermate WT mice [44]. The absence of PCSK9 did not determine significant changes in cholesterol and triglyceride concentrations in the liver, compared with WT mice, while the plasma cholesterol level in the PCSK9 null mice was 48% lower than those of WT with no difference in triglycerides. Although normal mouse plasma contains very little apoB-containing lipoproteins (VLDL or LDL) and the majority of circulating cholesterol is associated with HDL, the lack of PCSK9 determines a further reduction of the LDL cholesterol levels and a 30% decrease of HDL [44]. The effect of PCSK9 on HDL fraction is most likely due to the presence of apoE in mouse HDL that mediates the binding to the LDLR [44]. By crossing the PCSK9 null mice with the LDLR null mice, the changes of lipid profile were lost, indicating that the effect of PCSK9 on cholesterol homeostasis is dependent from the LDLR [19]. Moreover, the PCSK9 null mice express a 2.8-fold higher levels of hepatic LDLR than the WT mice and have a significant faster clearance ^25^I-LDL compared to WT. Consistently with previous work with PCSK9 overexpressing mice [5], the deletion of PCSK9 was associated with a slight reduction in the secretion of apoB48 and apoB100 [44]. The liver-specific PCSK9 null mice exhibit 27% lower level of plasma cholesterol compared to 42% of the complete knock-out mice, indicating that extrahepatic sources of PCSK9 may account for almost one-third of the regulation of cholesterol homeostasis [19]. 

The opposite approach was also described with the generation of transgenic mice overexpressing PCSK9 under the control of the apoE promoter and a liver-specific enhancer [19, 31]. Consistently with the results obtained by adenoviral overexpression of PCSK9 [5, 43], the transgenic overexpression of PCSK9, at plasma levels ranged from 146 to 440 *μ*g/mL, eliminated hepatic LDLR protein expression associated with higher LDL cholesterol levels and a minor effect on HDL [19, 43]. 

WT mice fed a chow diet are not susceptible to atherosclerosis, and this has led investigators over the years to develop three primary mouse models for examining atherosclerosis: (1) the high cholesterol diet (15% fat, 1.25% cholesterol, and 0.5% cholic acid), where lesion sizes remain small; (2) LDLR null causing a modest increase in plasma LDL cholesterol levels, where, to induce atherosclerosis, mice must be fed with a Western-type diet; (3) apoE null mice lack the primary apolipoprotein required for the uptake of lipoproteins through the hepatic receptors, leading to a greater plasma cholesterol increase than the LDLR null mice, and consequently develop atherosclerosis under regular chow diet, and advanced disease when the mice are maintained on a Western-type diet. Transgenic mice overexpressing PCSK9 induce a significant increase of LDL cholesterol resembling the phenotype of LDLR null mice [28]; thus, it is conceivable to hypothesize a possible effect on atherosclerotic plaque development. To address this hypothesis, Herbert et al. have recently generated transgenic mice expressing, at levels comparable to endogenous PCSK9, either WT or the gain of function mutant D^374^Y PCSK9 [108]. In particular, for the study it was selected two different strains of D^374^Y PCSK9 transgenic mice expressing low and high levels of exogenous PCSK9. When fed a chow diet, the three transgenic mouse lines showed virtually identical lipid profiles, with a slight increase in total cholesterol in the apoB-containing LDL fraction compared to control animals. However, the high cholesterol diet induced a more atherogenic lipid profile in both D^374^Y lines, with large increases in apoB- and apoE-containing lipoproteins. Associated to the atherogenic lipid profile observed in both low and high D^374^Y PCSK9 mice, it was observed the development of many lesions throughout the aortas, while no significant effect was seen in PCSK9 WT mice. These results strongly correlate with the plasma levels of proatherogenic LDL cholesterol, although positive effect of PCSK9 on triglyceride-rich lipoprotein from the liver has also been suggested [108]. 

More recently the role of PCSK9 on atherogenesis was evaluated in all three different models described above, such as in C57BL/6 WT mice fed Western diet, apoE^−/−^ mice, and LDLR^−/−^ mice [109]. The first analysis was conducted by comparing the C57BL/6 mice with PCSK9 null, and transgenic mice for PCSK9 fed Western-type diet (34% sugar, 21% fat, and 0.2% cholesterol). After 12 months of diet, the PCSK9 null mice had a 35% reduction in plasma cholesterol levels, while the transgenic mice showed 80% increase of cholesterol level primarily associated to LDL fraction. Histological analysis of atherosclerotic plaque demonstrated that only the transgenic mice developed measurable atherosclerotic plaque, associated with a 2.3-fold increase of cholesterol ester accumulation. The opposite effect was instead observed with PCSK9 null mice, with a 74% reduction of aortic cholesterol ester accumulation reaching comparable levels usually observed in WT mice fed regular chow diet. These results are comparable to those observed in the previous study, conducted with transgenic mice overexpressing PCSK9 and PCSK9 D^374^Y, where the analysis was done after 15 weeks of high cholesterol diet [108]. 

Since mice fed hypercholesterolemic diet do not develop atherosclerotic lesions, the potential beneficial effect of the absence of PCSK9 could not be assessed. To address this aim the apoE null mice were crossed with either PCSK9 transgenic or null mice. These mice were fed regular chow diet for 6 months and the plasma cholesterol levels were determined. As expected, mice with apoE background show a large accumulation in both LDL and VLDL cholesterol, and the absence or overexpression of PCSK9 does not significantly affect the lipid profile. Nevertheless, the aortic cholesterol ester accumulation was reduced by 39% in PCSK9 null mice and increased by 137% in transgenic mice associated with a 207% atherosclerotic lesion size. Interestingly, the absence of PCSK9 does not affect the development of atherosclerotic plaque at the level of aortic valves and root, determined by histological analysis, in the apoE null background. 

Finally, the effect of PCSK9 was determined in LDLR null mice fed hypercholesterolemic diet for 3 months. The PCSK9 null and transgenic mice crossed with LDLR null mice show similar plasma cholesterol levels, FPLC profile, aortic cholesterol ester accumulation, and plaque size compared to LDLR^−/−^ mice. These data indicated that the effect elicited by PCSK9 on cholesterol homeostasis and atherosclerotic plaque development is almost entirely dependent by the presence of LDLR [108]. However, the influence of PCSK9 on atherosclerotic plaque composition, rather than the size, still needs to be addressed and it will be more indicative of the potential atheroprotective effect on therapies against PCSK9. Of note, we have recently reported that PCSK9 is present in human atherosclerotic plaques which may locally regulate the expression of LDLR in macrophages [26]. 

Of interest, resistin, a small protein secreted by macrophages in humans and adipocytes in rodents [110], was shown to induce PCSK9 expression in hepatocytes and to reduce LDLR levels [111], suggesting that resistin-mediated upregulation of PCSK9 may contribute to the dyslipidemia commonly observed in obesity and diabetes, which is often characterized by altered levels of all lipid parameters. PCSK9 expression is also dependent by the action of insulin through the activation of SREBP-1c [112]. In diabetic rats it was also shown an altered expression of PCSK9 [113], and in hyperinsulinemic obese mice, the regulation of LDLR has been shown to be under the control of PCSK9, via the involvement of the mammalian target of rapamycin complex 1 (mTORC1) kinase activity [114]. In the same model, the transcriptional factor hepatocyte nuclear factor 1*α* (HNF1*α*) was demonstrated to be responsible for the regulation of PCSK9 levels [114]. These data are consistent with previous work demonstrating that the HNF1 binding site adjacent to sterol responsive element (SRE) is the critical regulatory sequence motif, and HNF1*α* is the predominant working partner for SREBP2 in the regulation of PCSK9 gene [115]. Taken together, PCSK9 expression appears to be deregulated under different pathological conditions such as diabetes and obesity, and potentially involved in the associated dyslipidemia. 

## 7. Emerging Evidences for an Extrahepatic Function of PCSK9

Although the majority of the studies have been focused on the regulation of PCSK9 on hepatic LDLR, it should be considered that mRNA PCSK9 is detectable in many extrahepatic tissues [3]. Surprisingly, PCSK9 significantly affects the LDLR expression in the liver but only marginally interferes with kidney and adrenal LDLR [116, 117]. This might be due to a lower local concentration of PCSK9 or to a different expression of cofactors required for PCSK9-dependent LDLR degradation. Nonetheless, the lack of effect of PCSK9 on LDLR in tissue that expresses reasonable amount of PCSK9 implies possible additional functions of PCSK9 beyond the regulation of cholesterol homeostasis. 

 PCSK9 was detectable in mouse and human isolated pancreatic islets, where only somatostatin positive *δ*-cells and not *α*- and *β*-cells express PCSK9 [23]. These cells apparently do not secrete detectable levels of PCSK9, although they respond to exogenously added PCSK9. Thus, the LDLR in pancreatic islets appears to be regulated, almost exclusively, by circulating PCSK9 of hepatic origin [23, 118]. The presence or absence of PCSK9 does not influence the insulin secretion assessed by incubating the pancreatic islets with low and high glucose concentrations [23]. Moreover, the PCSK9 deficiency did not alter the insulin secretion *in vivo* under basal and streptozotocin-induced diabetes conditions [23]. Significantly higher levels of fasting plasma glucose were, instead, observed in 4-month-old PCSK9 null male mice compared to WT [23]. By oral glucose tolerance test a significant increase of insulinemia was observed in WT mice, while no changes were observed in PCSK9 null mice, implying a possible role of PCSK9 on glucose homeostasis [118]. Nevertheless it must be noticed that this effect was only observed in male and 4-month-old mice, thus further evidences are required to corroborate this function. 

 PCSK9 mRNA is clearly detectable in the small intestine and colon at similar levels to that detected in the liver [3, 24]. By immunohistochemical analysis it was observed that PCSK9 is present almost exclusively in the epithelial barrier of the human duodenum and ileum, both in enterocytes and goblet cells [24]. The measure of plasma triglyceride levels in fasted mice over a 4-hour period after intragastric bolus of olive oil clearly documented a strong attenuation of the postprandial triglyceridemia [24]. This effect was not attributable to fat malabsorption in PCSK9 null mice but more likely to a reduction of apoB and chylomicrons secretion. Moreover, the absence of PCSK9 determines an almost 200% more hepatic uptake of chylomicrons than control WT mice. The combination of these two factors, together with a possible role of intestinal LDLR in the reuptake of nascent particles, could account for the strong reduction of postprandial triglyceridemia observed in PCSK9 null mice. A pharmacological inhibition of PCSK9 may, thus, help to manage both the hypercholesterolemia and the postprandial triglyceridemia, two important risk factors of cardiovascular disease.

 Together with the lower postprandial triglyceride levels, the PCSK9 null mice were also shown to accumulate abdominal fat, suggesting a role in the adipogenesis [22]. A detailed analysis demonstrated that PCSK9 null mice exhibited larger perigonadal (+70%) and perirenal (+90%) depots than WT mice, with an average increase of 2.8-fold of adipocyte volume [22]. This phenotype was demonstrated to be independent by the LDLR but rather to be mediated by the VLDLR targeted by PCSK9 [68]. From the analysis of different mouse models it was concluded that circulating, rather than local PCSK9, is responsible for adipose VLDLR protein regulation. 

By *in situ* hybridization analysis during embryonic development it has been shown that PCSK9 is expressed at E12.5 in the liver, small intestine, and telencephalon. Although PCSK9 was shown not to cross the blood brain barrier [117], its local expression in the frontal cortex regulates the LDLR levels but does not appear to dramatically alter the development of cerebellum [3, 119]. PCSK9 is present in the cerebellum also in the postnatal day 7, mainly localized in the external granular layer which regulates LDLR expression. These evidences suggest a possible functional role of PCSK9 in the cholesterol homeostasis of the brain [3]. In adult brain, PCSK9 mRNA is only expressed in the rostral extension of the olfactory peduncle, where the LDLR is poorly expressed [3]. By comparing the WT and null mice it was observed that the presence of PCSK9 does not affect the LDLR expression and the gross organization of the rostral extension of the olfactory peduncle and the olfactory bulb structures [119]. In an experimental model of brain injury, as shown also after partial hepatectomy [19], after 24 h and 72 h postinjury, PCSK9 mRNA was upregulated in the ipsilateral lesioned side of the dentate gyrus and disappeared after 1 week [119]. Mouse behavior and brain lesion size after injury did not differ between WT and PCSK9 null mice, and *de novo* neurogenesis in the dentate gyrus following transient middle cerebral artery occlusion was not altered in the absence of PCSK9. Nevertheless, the absence of PCSK9 has a relevant impact on LDLR expression in the lesioned side of the brain after ischemic stroke [119]. 

 Since VLDLR and ApoER2 are highly expressed in the central nervous system and have been previously implicated in Alzheimer's disease [120–124], the role of PCSK9 has been recently investigated *in vivo* [125]. PCSK9 was confirmed to be able to bind VLDLR and ApoER2 [68], but the analysis of PCSK9 null mice shows no effect on the steady-state level of LDLR family members in the adult mouse brain [125]. Although a previous study has shown that PCSK9 affects the expression of the *β*-site amyloid precursor protein-(APP-) cleaving enzyme 1 (BACE1), a membrane protease responsible for the production of toxic *β*-amyloid peptides that accumulate in neuritic plaques of Alzheimer's disease brains [126, 127], the analysis conducted in PCSK9 null and transgenic mice did not confirm these evidences [125]. 

Taken together, the current evidences on the role of PCSK9 in the central nervous system suggest that PCSK9 inhibition should not interfere with brain development and morphology or with brain recovery/damage after an ischemic stroke. PCSK9 therapies for coronary heart diseases are also likely not to be hampered by potential central nervous system effects. 

## 8. Therapeutic Approaches for Inhibiting PCSK9

PCSK9 has gained attention as a pharmacological target for treating atherosclerosis right after its discovery for at least three reasons:

1. PCSK9 directly downregulates the expression of hepatic LDLR which, in turn, raises plasma LDL cholesterol levels, a major determinant of cardiovascular disease risk [99].
2. Any specific inhibition of PCSK9 appeared to be safe since both loss of function mutations and women completely deficient in PCSK9 have been shown to be healthy without any other apparent metabolic defect [41, 75, 128].
3. The combination of PCSK9 inhibitors with statins should result to an additive, or even synergistic, hypocholesterolemic effect [44, 59, 76, 129].

Although several options can be theoretically pursued for inhibiting PCSK9 [130], currently, the most advanced therapeutic approaches are based on monoclonal antibodies (mAb), nonetheless also modified antisense oligonucleotides, short interfering RNA (siRNA), small molecules, peptides, and vaccines are under development (Table 2).

The mAb SAR236553/REGN727, first described with the name of mAb1 [131], is now in phase II. The REGN727 is a highly specific, fully human mAb to PCSK9 that interacts with a large number of amino acid residues of the catalytic domain and partially with the prodomain of PCSK9. By comparing the PCSK9:Fab1 complex of the mAb1 with the structure of PCSK9 bound to the EGF-AB domain of the LDLR [65], it has been postulated that Fab1 and the EGF-A domain bind to adjacent sites on PCSK9 and are sterically hindered from simultaneously binding to the PCSK9 protein [131]. Thus, by blocking the interaction of PCSK9 with the LDLR, mAb1 inhibits PCSK9-mediated degradation of the LDLR. The iv. injection of mAb1 (10 mg/kg) in C57BL/6 mice determined a significant reduction of total cholesterol levels by approximately 20–28% from 24 h to 144 h postadministration. mAb1 treatment increased hepatic LDLR protein levels by as much as 2.3-fold relative to control animals [131]. No changes in cholesterol levels were observed after injection of mAb1 in LDLR null mice, indicating that the presence of LDLR is required for the cholesterol lowering effect of mAb1. In nonhuman primates, mAb1 led to a statistically significant lowering of serum total cholesterol as early as 3 days after injection, with a maximal lowering of 48% ± 4% at day 10 compared to control animals. The effect of LDL cholesterol levels was even more striking with a maximum decrease of 80% ± 3% at day 10. Serum triglyceride levels were not altered by mAb1. In phase I of clinical trial, the effect of REGN727 was assessed in three separate studies [132]. Two of single doses were administered intravenously or subcutaneously in healthy volunteers and the third in a multiple-dose administered subcutaneously [132]. After single administration of intravenous or subcutaneous REGN727 it was observed a dose-dependent LDL cholesterol reduction in terms of both entity and duration of the effect. The higher doses of both intravenous (12 mg/kg) and subcutaneous (250 mg) determined approximately a 60% reduction of LDL cholesterol that last up to 64 and 30 days, respectively [132]. In the multiple-dose study, REGN727, administered subcutaneously, reduced the LDL cholesterol by 39.2% (50 mg), 53.7% (100 mg), and 61.0% (150 mg) in atorvastatin-treated patients. The most striking result of this study is that the LDL cholesterol response was similar in all subjects, regardless of whether they had familial or nonfamilial hypercholesterolemia or whether they were treated with atorvastatin or with a modified diet alone. In particular, the effect of REGN727 and atorvastatin in lowering LDL cholesterol appeared to be additive, not synergistically, since mean percent reduction was similar when REGN727 was administered alone or in subjects already receiving atorvastatin. The effect of REGN727 appeared also to be quicker than statins with a maximum reduction of LDL cholesterol within 2 weeks, whereas statins typically take longer [145]. The different mechanisms of action may account for the more rapid effect of REGN727. Differently from statins and bile acid sequestrants, REGN727 was seen to reduce, although not significantly in all doses, the lipoprotein(a). Finally, the administration of this mAb anti-PCSK9 appeared to be safe since no apparent drug-related adverse events were observed. 

 A second clinical trial, conducted in patients under atorvastatin treatment, essentially confirmed the previous results with a dose- and regimen-dependent LDL cholesterol reduction after subcutaneous administration [134]. The LDL cholesterol with 100 mg and 150 mg every two weeks was greater than with 200 mg and 300 mg every four weeks at week 12. As in the phase I trial [133], also in this trial it was observed a trend towards HDL cholesterol and apo-A1 increases [134]. Again, REGN727 increased the levels of lipoprotein(a) by 13% to 29% across the “every two week” regimens. The reason and the real effect on this parameter will require additional studies in order to be confirmed and understood, but it is possible that, with the large LDL cholesterol reductions, remaining competition from Apo-B and LDLR is minimal, enabling LDLR uptake of the lower-affinity Apo-B on lipoprotein(a). The LDL reductions of REGN727 were unaffected by atorvastatin dose indicating that, although both statin and PCSK9 inhibitors upregulate LDLR, their mechanisms of LDL cholesterol reduction are independent. 

Experimental studies have shown that statins may increase PCSK9 levels [44, 146, 147], however, this effect is not always observed in clinic [59, 76, 129]. Differences in study design could explain these apparent discrepancies. For instance, in the treating to new targets (TNT) trial, where no significant increase of PCSK9 levels was observed after treatment with 10 and 80 mg of atorvastatin, all the patients enrolled had a history of CHD. It is therefore possible that their statin-naïve plasma PCSK9 levels, a parameter that was not measured in that study, were on average higher than those of the normolipidemic individuals or of the asymptomatic patients enrolled in other studies [87, 148]. Plasma PCSK9 is also well established to positively correlate with LDL cholesterol in humans [20, 87, 149, 150]. In patients under statin therapy, this correlation is lost due to the simultaneous upregulation of LDLR and PCSK9 expression; this lack of correlation was observed in the TNT study [59]. Interestingly, existing evidences indicated that there is no significant inverse correlation between baseline PCSK9 and changes in LDL cholesterol in response to statin [20, 148]. The plasma levels of PCSK9 measured before starting statin therapy appear to be predictive of future cardiovascular outcomes only in patients maintained on 10 mg atorvastatin but not in patients uptitrated to receive 80 mg [59]. These may have a potential implication for the future development of PCSK9 inhibitors, where their use on top of statin could be beneficial for patients in whom increase of statin dose is not feasible. 

 Although larger and long-lasting trials are required to determine the frequency and severity of any possible side effects of REGN727, from these short studies it appears to be well tolerated. Particularly, it was not observed a significant increase in either hepatic or muscle-related enzymes. The major concern in terms of compliance is the evaluation, in larger clinical trials, of the real frequency of injection-site-reaction which was generally mild, transient, and nonprogressive [134]. 

A phase II clinical randomized controlled study with REGN727 was then conducted in patients with familial hypercholesterolemia and a concentration of LDL cholesterol of 2.6 mmol/L or higher [132]. Patients were randomly assigned to receive REGN727 150 mg, 200 mg, or 300 mg every 4 weeks, or 150 mg every 2 weeks, or placebo every 2 weeks. Subcutaneous administration of REGN727 resulted in mean reduction of LDL cholesterol of 28.9–67.9% (from 150 mg every 4 weeks to 150 mg every 2 weeks) of LDL cholesterol from baseline to week 12, compared with a 10.65% reduction with placebo. The best maintenance of the LDL cholesterol lowering effect was observed in the every 2 weeks regimens compared to every 4 weeks. Only the group of 150 mg every 2 weeks dose significantly increased the HDL cholesterol level by about 12.3%. Also in this study REGN727 therapy was well tolerated with only one patient in the 300 mg every 4 weeks group that terminated the study for an injection site reaction and generalized pruritus which was identified as being related to the study drug. No increases of more than three times the upper limit of normal in hepatic transaminases, or creatinine kinase, was observed.

 The ability of REGN727 at 150 mg every 2 weeks to achieve a mean LDL cholesterol of about 1.3 mmol/L, a further decrease greater than 2.6 mmol/L from that of high-dose statin plus ezetimibe treatment, and to result in more than 80% of patients treated to achieve an LDL cholesterol concentration lower than 1.8 mmol/L support the validity of this new therapeutic opportunity for the treatment of patients with familial hypercholesterolemia.

 Beyond the REGN727, at least other two pharmaceutical companies (Pfizer Inc. and Merck) are currently developing mAb directed to PCSK9 [136–139]. In particular, in Merck Research Laboratories it has been selected and studied an antibody to the C-terminal domain of PCSK9 [138]. The relevance of this domain has been only recently appreciated [67], and the use of this pharmacological tool has helped to elucidate the mode of action of PCSK9 on LDLR [138]. Both human Fab and IgG2, 1G08, bind purified recombinant PCSK9 with an affinity of 550 pM. Fab 1G08 only marginally affected (50–60%) the cellular LDL uptake and only in the presence of exogenous PCSK9, while the IgG2 was not effective. More interestingly, the alteration of LDL uptake by Fab 1G08 was not due to the inhibition of the PCSK9 binding to the receptor. Instead, the inhibitory effect of 1G08 on PCSK9 cellular function appears to be mediated, at least in part, by the attenuation of cellular PCSK9 uptake. Although 1G08 Fab significantly affects PCSK9 function through a new and yet unclear mechanism of action, its clinical interest does not appear to be high especially because the IgG form, with favorable pharmacokinetic properties, was shown to be less effective than the Fab [138]. 

A more conservative approach for inhibiting PCSK9 was also developed by Merck with the selection of mAb directed to the catalytic domain of PCSK9 including the entire LDLR EGF-A binding site [137]. The effect of this antibody was first assessed in a transgenic mouse model expressing both human CETP and human LDLR (CETP/LDLR-hemi). In these animals the plasma lipid and PCSK9 profiles mimic the human situation, and 1D05-IG2 reduces, dose-dependently, plasma LDL cholesterol. A single injection of 4 mg/kg reduced the level of LDL cholesterol by 50% at 48 h postdose. The *in vivo* efficacy of 1D05 was also evaluated in rhesus monkeys. In this animal model, a single i.v. injection of 3 mg/kg led to a decrease of up to 50% in circulating LDL cholesterol. The effect was observed within 24 h of dosing and lasted over 2 weeks. 

A second antibody that binds to PCSK9 with high affinity disrupting the PCSK9-LDLR interaction was then identified and developed from the same company [139]. The 1B20 antibody binds to human, rat, rhesus, and mouse PCSK9 with high affinity at *K*_*D*_ of 0.3, 4.5, 0.6, and 1 nM, respectively. A single 1.1 mg/kg i.v. dose of 1B20 induced a 29% LDL cholesterol reduction 48 h postdose in CETP/LDLR-hemi mice. As expected, the 1B20 induced a sustained increase of hepatic LDLR expression [139]. A multidose study in CETP/LDLR-hemi mice was performed using either 3 or 10 mg/kg doses of 1B20. Administration of 1B20, 14 days apart, determined a robust 50–70% LDL cholesterol-lowering effect. 

Since the subcutaneous administration is the preferred route of delivery in humans, a study was conducted in healthy rhesus monkeys to evaluate pharmacokinetic and pharmacodynamics of 1B20 following a single subcutaneous or intravenous dose of 1 and 10 mg/kg [139]. Similar serum 1B20 levels were observed between subcutaneous or intravenous at dose of 1 mg/kg, while higher levels were observed in the subcutaneous dose 10 mg/kg. In agreement with the pharmacokinetic profile, the 1 mg/kg dose determines a similar LDL cholesterol-lowering action, while 10 mg/kg of subcutaneous administration was more potent than the intravenous dose. Thus, a direct correlation between the pharmacokinetic and pharmacodynamic profile was observed [139]. 

Finally, the hypocholesterolemic effect of 1B20 was assessed in a cohort of metabolic syndrome monkeys in combination with statin treatment. The combination of 1B20 and simvastatin reduced LDL cholesterol more than simvastatin or 1B20 alone, highlighting the advantage of an anti-PCSK9 antibody and statin combination therapy over either treatment alone [139]. 

Of interest, in both mouse and monkey studies [139], 1B20 treatment led to decreases in plasma-free (unbound) PCSK9 levels and increases in plasma total PCSK9 levels. This effect is probably due to a sequestration of PCSK9 by 1B20, which blocks PCSK9 uptake and clearance by the liver, the major organ for PCSK9 clearance in the body. Similar results were also observed with the 1D05 antibody [137] increasing, to a lesser extent, the total plasma PCSK9. Understanding the basic molecular mechanisms governing the clearance of mAb to PCSK9 may help to ameliorate their pharmacokinetic profile. It is important to mention that the fast clearance of PCSK9 mAb is probably due to a short half-life time of PCSK9 in the circulation, approximately 5 min [151]. This rapid clearance seems to be only partially dependent by the LDLR, since the half-life increases to 15 min in LDLR null mice [151]. More recently, the clearance of PCSK9 through an LDLR-independent mechanism has been hypothesized by a direct comparison of familial hypercholesterolemia homozygotes, heterozygotes, and normocholesterolemic subjects [152]. 

Pfizer Incorporation is also developing a humanized mAb directed to the LDLR binding domain of PCSK9 [153]. The J16 antibody resulted from a screening for anti-PCSK9 mAb among hybridoma cell lines generated by fusing the spleen of various WT and PCSK9 null mice immunized with recombinant human PCSK9 protein. From this first screening it was selected the J10 antibody that was further engineered into a human IgGdeltaA [154] and *κ* chain antibody with an improved antigen binding affinity compared to the original J16 antibody [153]. The resulted human antibody binds to recombinant human PCSK9 with a *K*_*D*_ of approximately 5 pM, to cynomolgus monkey with *K*_*D*_ less than 100 pM, and to mouse PCSK9 with *K*_*D*_ of 35 pM.

When 10 mg/kg J10 was administered, as a single intraperitoneal dose, to C57BL/6 mice fed a normal chow diet, serum total cholesterol and HDL cholesterol levels were maximally reduced by 46% and 43%, respectively, at day 7, with no effect on triglyceride levels [153]. In addition, the LDLR-dependent effect of J10 was demonstrated by using LDLR null mice, where no effect on lipid profile was observed after intraperitoneal injection of 10 mg/kg of J10. Importantly, mice fed a high fat diet (60% kcal fat) or high fat/high cholesterol diet (40% kcal fat with 2.8 mg/kcal cholesterol) were completely resistant to J10. To further investigate these findings, the effect of a single injection of 3 mg/kg J16 in hypercholesterolemic nonhuman primates was investigated. A 64% reduction of LDL cholesterol was observed by day 3 posttreatment, a very similar effect to that observed in monkeys fed normal chow diet. Moreover, the effect of J10 was shown to be additive to statin treatment in hypercholesterolemic nonhuman primates [153]. 

Taken together several pharmaceutical companies are currently investing resources on mAbs against PCSK9 with similar mechanism of action, although some differences in terms of potency are observed, all of them appear to be promising for a future clinical development. 

Since PCSK9 is highly expressed in the liver, and its primary function is to degrade LDLR in hepatocytes, it is an ideal molecular target for the antisense oligonucleotide technology. As a proof of concept of this pharmacological approach, a first series of experiment was carried out by using an antisense oligonucleotide complementary to the mousePCSK9 gene and administered in hyperlipidemic mice [140]. The intraperitoneal injection of a water soluble chimeric 2′-O-methoxyethyl phosphorothioate 20-mer antisense oligonucleotide (ISIS 394814; 50 mg/kg twice weekly) to high fat-fed C57BL/6 mice reduced by 92% the PCSK9 mRNA levels after 6 weeks of treatment [140]. This effect was associated with a significant increase by 2.7-fold of hepatic apoliprotein B mRNA editing enzyme catalytic polypeptide 1 (apobec-1) mRNA and with no changes on other key cholesterol and fatty acid biosynthetic genes. The effect on apobec-1 by the antisense ISIS 394814 increased the serum apoB-48 protein levels and reduced apoB-100 levels without affecting the apoA-I. The reduction of PCSK9 expression determined a 2-fold increase of hepatic LDLR and a reduction of circulating total cholesterol and LDL cholesterol by 53% and 38%, respectively. Differently from mAbs PCSK9 inhibitors, in this study it was observed a significant 65% reduction of hepatic triglyceride levels. Similar effect was previously reported in PCSK9 null mice [44]. Consistent with the proposed mechanism of PCSK9, no effect on plasma cholesterol levels was observed in LDLR null mice after administration of antisense ISIS394814. 

 The second approach utilized liposome encapsulated small interfering RNAs siRNAs as injectable lipidoid nanoparticles (LNP) [143]. Various doses of the lipidoid-formulated PCS-A2 were injected via the tail vein into mice and rats. The maximal PCSK9 mRNA silencing was achieved after administration of 5 mg/kg of PCS-A2 with a reduction of PCSK9 mRNA by about 60–70%, which translated into a 30% lowering effect on total plasma cholesterol levels. The lipid-lowering effect persisted for approximately 20 days after a single injection with the highest dose utilized (7.5 mg/kg). Differently from the antisense approach [140], the siRNA directed to PCSK9 did not alter the hepatic triglyceride levels in mice [143]. This approach was also validated with the PCS-C2 directed to human PCSK9 in mice expressing human PCSK9 cDNA under the apoE promoter [31]. A single dose of 5 mg/kg of PCS-A2 or PCS-B2 resulted in a significant reduction of LDL cholesterol beginning at day 3 after the dose until day 14 (for PCS-A2) and day 21 (for PCS-B2) in nonhuman primates [143].

The final “silencing” strategy was to utilize high affinity, single stranded, unformulated, 12–16 nucleotide short locked nucleic acid (LNA) modified gap-mer antisense oligonucleotides [141, 142]. This approach does not require formulation, and it has been tested in mice. Intravenous administration determined a 60% reduction of PCSK9 mRNA in the liver, that lasted more than 16 days, associated with two fold increase of hepatic LDLR. The estimated ED_50_ was 9 mg/kg [142]. The same technology was then translated in non-human primates [141]. Also in this case it was observed a very long lasting effect of LNA oligonucleotides with a maximal 50% reduction of LDL cholesterol level after 21 days after injection and a significant effect until day 56 [141]. The effect of two oligonucleotides, SPC5001, and SPC4061 was then examined in a multiple-dose study, comprising an initial loading dose of 20 mg/kg, followed by four weekly maintenance doses of 5 mg/kg. Both inhibitors, administered subcutaneously as unformulated molecules in buffer saline, produced a significant reduction of serum PCSK9 protein levels (−85% for SPC5001) maintained for all durations of the study. The LDL cholesterol-lowering effect was on average of 50% up to 70% for SPC5001, while SPC4061 was less effective. Liver LDLR protein levels, analyzed by western blot analysis, increased by 67% in SPC5001treated monkeys compared to saline controls, and there was no evidence of cholesterol accumulation in the liver [141]. 

An alternative approach for inhibiting the PCSK9 LDLR interaction may be the use of small peptide mimicking the EGF-A/B domain of LDLR [66, 70]. The validity of this approach has been also confirmed by using HepG2 cells overexpressing EGF-A [88]. Endogenous inhibitor of PCSK9 has also been described and potentially leading to the development of new pharmacological entities with an anti-PCSK9 functionality [155]. However, the peptide-based approach appears to be less effective than mAb for at least two reasons: first peptides have a substantial lower affinity than antibodies, unless this may not be an issue given the low affinity of PCSK9 for LDLR at neutral pH; second small peptides have usually demonstrated to have a low serum half-lives necessitating frequent dosing, and the oral administration is not suitable. The potential advantage of developing peptidic inhibitors remains a considerable cost advantage compared with antibodies.

 While the development of classic small molecule inhibitors that would interfere with PCSK9 LDLR interaction appears difficult, this type of pharmacological approach has been proposed, considering that the autocatalytic cleavage is required to transport PCSK9 from the endoplasmic reticulum to the Golgi apparatus and for the secretion of the mature protein. Thus, by blocking the catalytic activity of PCSK9, the small molecule would prevent protein processing and secretion affecting PCSK9 functionality. In order to achieve a proper pharmacological inhibition, the small molecule will need to reach the endoplasmic reticulum and to selectively inhibit PCSK9 leaving the other proprotein convertases functionally active. The recent description of a cell-based assay for the determination of PCSK9 catalytic activity will certainly help to identify new PCSK9 inhibitors [18], even if the absence of a crystal structure of the active site of unprocessed PCSK9 rends more difficult the design of this new class of pharmacological agents. The use of small molecule as potential class of PCSK9 inhibitors will be advantageous in terms of cost and route of administration. Moreover, the effects of a small molecule would likely be relatively short in duration as compared to the very long pharmacokinetic profile of both mAb and RNAi approaches; thus, any eventual side effects should be reversible in the short term, and a quick dose adjustment would be possible.

Finally, an interesting new approach that has been recently described involved the direct immunization against PCSK9 [144]. This approach is based on the findings that one single amino acid difference between the immunizing antigen and the endogenous protein is sufficient to elicit an immune response and the generation of antibodies which cross-react with endogenous protein and remove it from the circulation [156, 157]. Using this system it was observed a 2-fold increase of the hepatic LDLR and a 60% reduction in LDL cholesterol at day 14 after immunization in BALB/c mice. By ELISA assay it was shown that 14 days after immunization the concentration of circulating PCSK9 was reduced by 66%. This approach appears to be suitable for a specific inhibition of a particular target protein in an experimental setting. Further studies will be required in order to better characterize this approach in terms of safety, specificity, efficiency, and so forth before considering a human use. 

## 9. Concluding Remarks

From the data currently available, it is conceivable to conclude that, due to its function as a regulator of LDLR protein expression in the liver, PCSK9 represents the most promising pharmacological target for the new era of treatment of cardiovascular diseases. The enormous interest on this molecule is confirmed by a number of pharmaceutical companies currently involved in the development of PCSK9 inhibitor. However, the current approach suitable for inhibiting PCSK9 appears to be restricted to mAbs that block the LDLR-PCSK9 interaction. Obviously this therapeutic opportunity erased some concerns in terms of cost of the therapy and the compliance of the patient. Nonetheless, although parental administration is not particularly attractive for lifelong treatment, it could be accepted by high-risk patients who cannot achieve LDL cholesterol levels of 50 mg/dL on available combination therapies or who have limiting side effects to statins or other hypolipidemic agents. The target population that more likely will benefit from this type of therapy is patients with familial forms of hypercholesterolemia. 

 The challenge of identifying safe, oral active small molecule inhibitors of PCSK9 is definitely a future goal that should be pursued vehemently. Such inhibitors, in combination with statins, may be of great value in the treatment of hypercholesterolemia even in patients not at risk but with minor hypercholesterolemia. The time will tell us whether this kind of approach will be suitable for controlling the hypercholesterolemia.

 Finally, the antisense technology is certainly capable to inhibit a specific target with high selectivity, and preclinical data support the possibility to utilize this type of therapy for targeting PCSK9; however, very little is known regarding the safety profile of this approach. The most advanced oligonucleotide antisense for the treatment of dyslipidemia is mipomersen, an apoB synthesis inhibitor [158]. Mipomersen has been generally well tolerated and had an acceptable safety profile in the phase II and phase III studies. The most common adverse events are injection site reactions, flu-like symptoms, and increases in liver function tests. In particular, the injection site reactions, that occur in the majority of the patients (75%–100%) are considered an antisense class-related phenomenon also observed with other antisense drugs and likely also with anti-PCSK9 oligonucleotides. Although these reactions are not considered to be serious safety concerns but it may affect patient compliance.

 In conclusion, an important effort has been made in order to develop new PCSK9 inhibitors, and, in the future, therapies capable to inhibit PCSK9 expression (oligonucleotide antisense), processing (small molecules), and/or interaction with LDLR (mAbs) could represent a real benefit for the treatment of hypercholesterolemia and associated cardiovascular diseases. 

## Figures and Tables

**Table 1:** PCSK9 allelic variants with known functional property.

Functional domain	DNA allele	Mutation	Function	Ref.
5′UTR, 2.5-fold increase of transcriptional activity	C^−332^A		Gain	[71]
Signal peptide	G^10^A	Val^4^Ile	Gain	[72, 73]
Prodomain	G^94^A	Glu^32^Lys	Gain	[73]
Prodomain	A^161^C	Glu^54^Ala	Gain	[73]
Generation of a truncated peptide (Ala^68^fsLeu^82^X)	^ 202^del G	Ala^68^ProfsX15	Loss	[74]
Prodomain	C^230^T	Thr^77^Ile	Loss	[74]
Prodomain	C^277^T	Arg^93^Cys	Loss	[73]
Prodomain	^290–292^del GCC	Arg^97^del	Loss	[75]
Prodomain	C^310^T	Arg^104^Cys	Gain	[73]
Prodomain	G^316^A	Gly^106^Arg	Loss	[76]
Prodomain	T^341^C	Val^114^Ala	Loss	[74, 77]
Prodomain	T^381^A	Ser^127^Arg	Gain	[39, 78, 79]
Prodomain	G^385^A	Asp^129^Asn	Gain	[74]
Prodomain	A^386^G	Asp^129^Gly	Gain	[78]
Generation of a truncated peptide	C^426^G	Tyr^142^X	Loss	[80]
Catalytic	C^503^A	Ala^168^Glu	No effect	[78]
Catalytic	G^644^A	Arg^215^His	Gain	[79]
Catalytic	T^646^C	Phe^216^Leu	Gain	[39]
Catalytic	A^654^T	Arg^218^Ser	Gain	[81]
Catalytic	C^655^G	Gln^219^Glu	Loss	[73]
Catalytic	G^706^A	Gly^236^Ser	Loss	[79]
Catalytic	C^716^A	Ala^239^Asp	Loss	[73]
Catalytic	C^757^T	Leu^253^Phe	Loss	[82]
Catalytic	A^1061^T	Asn^354^Ile	Loss	[79]
Catalytic	G^1070^A	Arg^357^His	Gain	[81]
Catalytic	G^1120^T	Asp^374^Tyr	Gain	[40, 83, 84]
Catalytic	G^1120^C	Asp^374^His	Gain	[85]
Catalytic	C^1171^A	His^391^Asn	Loss	[82]
Catalytic	A^1274^G	Asn^425^Ser	Gain	[82, 86]
Catalytic	G^1284^A	Trp^428^X	Loss	[73]
C-terminal domain	C^1300^T	Arg^434^Trp	Loss	[87]
C-terminal domain	G^1355^A	Gly^452^Asp	Loss	[73]
Disrupts normal folding of the C-terminal domain	T^1384^C	Ser^462^Pro	Loss	[79]
C-terminal domain	C^1405^T	Arg^469^Trp	Gain	[81, 82]
C-terminal domain	C^1486^T	Arg^496^Trp	Gain	[86]
C-terminal domain	G^1540^A	Ala^514^Thr	Gain	[73]
C-terminal domain	G^1564^A	Ala^522^Thr	Gain	[74]
C-terminal domain	A^1658^G	His^553^Arg	Gain	[82]
C-terminal domain	C^1660^G	Gln^554^Glu	Loss	[82]
C-terminal domain	C^1847^T	Pro^616^Leu	Loss	[74]
C-terminal domain	G^1870^A	Val^624^Met	Gain	[73]
C-terminal domain	C^2004^A	Ser^668^Arg	Loss	[73]
Generation of a truncated peptide retained in the ER	C^2037^A	Cys^679^X	Loss	[80]

**Table 2:** Pharmacological approaches currently under development against PCSK9.

Approach	Name	Company	Ref.
mAb to LDLR binding domain of PCSK9	REGN727	Regeneron Pharmaceuticals Inc.	[131–134]
mAb to LDLR binding domain of PCSK9	mAb1	Amgen Inc.	[131]
mAb to LDLR binding domain of PCSK9	AMG 145	Amgen Inc.	[135]
mAb to LDLR binding domain of PCSK9	J16	Pfizer Inc.	[136]
Fab and IgG2 to catalytic domain	1D05	Merck Research Laboratories	[137]
Fab to C terminal domain	1G08	Merck Research Laboratories	[138]
mAb to LDLR binding domain of PCSK9	IB20	Merck Research Laboratories	[139]
Antisense oligonucleotide	ISIS 394814	Isis Pharmaceuticals, Inc.	[140]
Antisense oligonucleotide	SPC5001 SPC4061	Santaris Pharma A/S	[141, 142]
siRNA	PCS-A2/B2/C2	Alnylam Pharmaceuticals	[143]
Vaccine		Merck Research Laboratories	[144]
